# Students’ Aid Series

**Published:** 1885-12

**Authors:** 


**STUDENTS' AID SERIES.—Aids to Surgery.** By GEORGE BROWN, M.R.C.S., L.S.A., late Demonstrator of Anatomy at Westminster Hospital Medical School, etc. New York and London: G. P. Putnam's Sons. 1885.

The price of these "Aids to Students" is so low that every student can well afford to buy each of the thirteen, and in so doing will be able to review the subjects, and in addition get many points that in reading the text-books may have escaped his notice. This number is especially good.

**Aids to Medicine.** Part III. Double Part. Diseases of the Brain and its Membranes; of the Nervous System; of the Spinal Cord and of the Ear. By C. E. ARMAND SEMPLE, B. A., M. B., Cantab, L. S. A., M. R. C. P., London, Physician Northeastern Hospital for Children, Hackney, etc.

A work on the subject of nervous diseases so elementary in character is especially useful, both to the physician and student. To the physician who may have had but little opportunity to study this branch, to the student who for the first time is taught it, the book contains a good review of the subject.

